# Translation and cultural adaptation of the Greek integrated palliative care outcome scale (IPOS): challenges in a six-phase process

**DOI:** 10.1186/s12904-023-01278-2

**Published:** 2023-11-02

**Authors:** Despina Anagnostou, Stylianos Katsaragakis, Irene Panagiotou, Elisabeth Patiraki, Aliki Tserkezoglou

**Affiliations:** 1 Kyoto University, Kyoto, Japan; 2 National and Kapodistrian University of Athens, Athens, Greece; 3 National Primary Care, Athens, Greece; 4 Galilee Palliative Care Unit, Spata, Attica Greece

**Keywords:** Outcome measures, IPOS, Palliative care, Cross-cultural adaptation, Cognitive interviews, Greek

## Abstract

**Aim:**

To translate and culturally adapt IPOS to the Greek population.

**Methods:**

A four phases- sequential study, which included verification of conceptual equivalence, double forward- backward translations and conceptual cognitive debriefing. Focus group interviews used ‘think aloud’ and ‘verbal probing’ techniques. Interviews were audio-recorded, transcribed verbatim and thematically analyzed using predefined categories. Purposely sampled from two oncology and palliative care units in Athens.

**Results:**

The Integrated Palliative Care Outcome Scale was well accepted by both patients and health professionals. Overall comprehension and acceptability of the scale were good. The comprehension and judgement challenges identified in the pre-final version were successfully resolved in the cognitive interviewing phase. Five out of the seventeen translated items of the scale were modified after cognitive debriefing. Comprehension difficulties were identified with specific terms (e.g., energy/feeling depressed) and with some answer options. Severity of symptoms and not their impact was a common difficulty. A judgement challenge was reported in relation to 7-days recall and fluctuation of symptoms. Layout concerns in relation to length of questions were also stated. All questions were considered important and none as inappropriate.

**Conclusion:**

This study demonstrated face and content validity and acceptability of the Integrated Palliative Care Outcome Scale in the Greek context. Cognitive Interviewing proved valuable in refining concepts within the specific cultural context.

**Clinical implications:**

The IPOS outcome measure tool is now being used routinely in a palliative care service in Athens and is currently used to evaluate service outcomes.

**Supplementary Information:**

The online version contains supplementary material available at 10.1186/s12904-023-01278-2.

## Background

Patient-reported outcome measures (PROMs) are validated questionnaires completed by patients to measure their perceptions of their own health status/wellbeing and are widely used to improve outcomes of health care services and quality of care [1]. Patient-reported outcome measures (PROMs) have become increasingly important in health care. Their value for quality improvement and their role in informing daily clinical care and in driving decision-making are particular advantages [2, 3]. The Integrated Palliative Care Outcome Scale (IPOS) is a patient-centred outcome measure developed after merging the Palliative care Outcome Scale and the Palliative care Outcome Scale-Symptoms [4, 5].

Patient-reported outcome measurement (PROMs) have been progressively endorsed by palliative and end-of-life (EOL) care. However, their application in both clinical practice and research is variable, with some measures being implemented only a few times [6]. Outcomes measures need to reflect the concerns of the patients’ complex needs but also minimize respondent burden [4]. The diversity of symptoms, the complexity of the problems and the ever changing needs and priorities of patients who navigate their disease trajectories are some of the challenges specific to EoL care which also influence the measurement of outcomes [7]. Consensus on outcomes and measures that can been used across different settings and countries, might support international collaboration to explore barriers and suggest solutions for bigger populations within the European and Global context. [8, 9]. The adaptation of the IPOS tool in Greek may contribute to the movement of establishing standard core outcome measures in palliative care and support initiatives for robust comparative research in the field [6, 10].

Palliative Care (provision) in Greece is rather fragmentary, both in terms of its scope and its accessibility to State funds. Greece is among the group of countries characterized by the scarcity of their hospice/specialized Palliative Care services [11]. Despite earlier recommendations from the Council of Europe [12] and the National Action Plan for Cancer 2011–2015 [13], there are still a few palliative care services. The European Study PRISMA highlighted the lack of national effort for evaluating outcomes in palliative care in Greece and underlines the need for adapting and validating quality indicator measurement instruments in the Greek language [12, 14]. This lack and need for a patient-reported outcome measure in the Greek context led to this study.

The aim of this, therefore, was to translate and cross-culturally adapt the Integrated Palliative care Outcome Scale (IPOS), to be used by the Greek population.

## Methods

### The instrument

Ten questions comprise the IPOS tool. The tool includes four versions (staff/ patient, including 3- & 7-days recall). It consists of seventeen items, incorporating aspects of physical symptoms (items 1–10); emotional symptoms (items 11–14); communication and problems (items 15–17. Each item is scored with a Likert scale (0–4) and can be measured either individually and /or in subscales. It also includes two open-ended questions, assessing any additional problems/ symptoms.

### Cross cultural translation

**Fig. 1:**
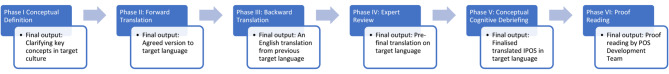
The six phases for achieving cross-cultural adaptation [15]

We followed the guidelines for the POS family of measures for the translation and cross-cultural adaptation [15]. These were built on well-established translation and validation standard and guidelines offered by organisations such as the European Organization for Research and treatment of Cancer- EORTC and the Mapi Institute [16–18] (Fig. 1).

### Phase I: Conceptual definition or equivalence

The aim of this phase was to clarify concepts and terms featured in the IPOS tool to ensure their equivalence in the Greek culture. We conducted a literature review on health-related quality of life studies in Greece with a focus on chronic ill patients and quality of life assessment. We searched for the available tools validated in Greek. We looked to identifying key concepts underlined in each item, similar terminology such as symptoms, and terms used in scaling, their meanings, and translations within health-related questionnaires and quality of life. Subsequent semi-structured interviews with five health care professionals (two doctors and three nurses) with experience in palliative care, and informal discussions with five palliative-care patients verified the terms which informed the Greek IPOS translation.

### Phase II: Parallel blind forward translation

The aim of the forward translation was to develop a tool equivalent between the original and Greek culture after consideration of meanings and interpretations of each item. The forward translation was conducted by two independent translators, one being a clinical doctor involved in palliative care and the other being a lay member, naïve to palliative care. We hoped that the second translator would be able to detect the more subtle differences in meaning to the original and offer a translation that reflects the language used by the public. Both translators were instructed to consider conceptual, semantic, and text-normative equivalence. They were also suggested to record alternative terms they considered. Comparisons between the translations, and identified discrepancies lead to discussion with a mediator which allowed for the best choice or words and a synthesis between the two versions as the final version of phase II.

### Phase III: Parallel blind backward translation

The backward translation aimed at validity checking and ensuring that the new translated version was accurate when compared with the original version [19, 20]. It was conducted by two independent translators following the same guidelines with the forward translation. They were both bilingual, one was a health care professional with experience in palliative care whilst the other was unfamiliar with the field. As with the forward translation, the mediator collected the two versions, compared them with the original, and reconciled any discrepancies with the help of the two translators.

### Phase IV Expert review

The Greek translation was reviewed by an expert review panel consisted of members of a palliative care service and the research team undertaking this study. It included all translators, two doctors and three nurses with knowledge in palliative care alongside the researchers. The panel met several times with the aim of evaluating, revising, and consolidating the instructions, items and response formats of the translated IPOS tool. The expert review outcome was the pre-final version of the IPOS Greek tool. The produced version was subsequently tested via conceptual cognitive debriefing.

### Phase V Conceptual cognitive debriefing

The purpose of conceptual cognitive debriefing was to test the new IPOS tool in the Greek language. We conducted two focused group interviews, with both palliative care patients and health care professionals respectively. Patient participants were purposively sampled via two different units, with the aim of achieving variation in relation to gender, age, diagnosis, education level and disease phase. Health care professional participants were selected with the aim to cover for different specialties and clinical experience in general or specialist palliative care.

Participants completed the questionnaire whilst using the ‘think aloud’ techniques, and concurrently discussing their answers and any problems regarding its completion [21, 22]. A combination of spontaneous questions generated during the interviews alongside an interview guide with probe questions was used (Additional file 1: Interview guide). The probing questions were based on the question response model by Tourangeau, addressing issues of comprehension, retrieval, judgement, and response formulation [23]. The focus group interviews were audio-recorded and transcribed verbatim. The Interview data was analysed using thematic analysis [24]. The analysis followed the pre-defined categories of the Tourangeau model in addition to layout, acceptability / questionnaire burden categories. Adjustments were considered to the pre-final version of the Greek IPOS because of the cognitive debriefing.

### Phase VI: Proof reading

The translated instrument was again reviewed by the research team and subsequently submitted to the POS development team at Cicely Saunders Institute to proofread and endorse before psychometric validation. After discussions and further modification, the final version of IPOS Greece was produced.

## Results

### Demographics

A total of 15 participants, nine health care professionals and six patients participated in the cognitive debriefing interviews between May 2018 and January 2019, excluding the four clinicians who conducted the focus group interviews. Participants were recruited purposively from a specialist palliative care outpatient/home care unit and a palliative day care unit, to cover for different palliative care needs as well as health care professionals’ experience in both general and specialist palliative care. The patient group covered variation in terms of gender, educational status, cancer diagnosis, setting of care provision and disease phase. Inclusion criteria for patients were age of > 18 years old, have palliative care needs, being able to provide informed consent, complete the IPOS tool and native speakers in Greek. The clinician group included 4 nurses, 2 physicians and 3 social workers with a varied experience in palliative care from one to seven years. The focus group interviews were 120–150 min long. Time to complete the IPOS tool ranged from 7.7 min to max 11.24 min in the patients’ group and 4,4–10 min in the HCP’s group. Participants’ characteristics are reported in Tables 1 and 2.

**Table 1:** Focus groups’ participants’ role and background

Profession	Place of work	Role in the study
Nurse	Palliative Day Care	Clinician
Social worker	Palliative Home Care	Clinician
Physician	Palliative Home Care	Clinician
Nurse	Palliative Day Care	Clinician
Social worker	Palliative Home Care	Clinician
Nurse	Palliative Home Care	Clinician
Nurse	Palliative Home Care	Clinician
Nurse	Palliative Home Care	Clinician
Social worker	Palliative Home Care	Clinician
Nurse	Oncology setting & Academia	Conductor of the focus group
Nurse	Academia	Co-conductor of the focus group
Physician	Oncology setting & Home Care	Conductor of the focus group
Physician	Palliative Home Care	Co-conductor of focus group

**Table 2:** Patients’ demographic and clinical data

Total number of patients		N (6)
Sex	Female	4
	Male	2
Age (years)	Median (min-max range)	75 (56–82))
Marital status	Married	3
	Widowed	3
Education	University / Technological	4
	Secondary education	2
Diagnosis	Breast cancer	3
	Lung cancer	2
	Colon cancer	1
Care Setting	Palliative home care	3
	Palliative day care	3
Disease Phase	Stable	4
	Unstable	2
IPOS overall score	Median (min- max range)	25 (16–40)
Time to IPOS completion	Median (min-max range)	10’3” (7’7”-11’24”)

### Phases I-IV

#### Phase I

We looked to identify differences throughout existing translations of IPOS terms contained in other questionnaires of quality-of-life and possibly palliative care in Greek, through a literature review. Twenty-two questionnaires translated from English to Greek were identified which included terms used in IPOS.

Some items were translated consistently across the different tools, such as pain (πόνος), nausea (*ναυτία*), vomiting (*έµετος-έµετοι*) and constipation (*δυσκοιλιότητα*). However, terms related to anxiety, depression, shortness of breath, sore or dry mouth and lack of energy received various translations with slightly different conceptual meanings (Table 3). The terms ‘poor mobility’ and ‘at peace’ were not identified in any of the retrieved tools.

**Table 3:** Alternative translations for IPOS items

IPOS item	Greek translations
Anxiety (feeling anxious)	*άγχος*’; concerned -‘*ανήσυχος*’; irritable- ‘*ευερέθιστος’;* tense-‘*σε ένταση’*
Depression (feeling depressed)	‘*κατάθλιψη/ καταθλιπτικά’;* sad/sadness - ‘*θλίψη*, *λυπηµένος/ λυπηµένα’*
Shortness of breath	να *κόβεται η ανάσα σας’*, ‘*δυσκολία στην αναπνοή*΄, ‘*γρήγορη αναπνοή’* ,‘*λαχάνιασµα*’; dyspnoea’- ‘*δύσπνοια*’
Weakness/ Lack of energy	*αίσθηµα αδυναµίας, εύκολη κόπωση, εξάντληση, µειωµένη ενέργεια’*
Sore or dry mouth	dry mouth- ‘*ξηρό στόµα’, ξηροστοµία;* mouth sores- ‘*στοµατικά έλκη/έλκη στόµατος*’

The alternative translations identified in the literature were further discussed within the next step of interviews with senior clinical members of the palliative care units. A conceptual definition meeting followed results from the interviews and included the views of the members of the IPOS team who were involved in translations of other tools. Some concerns were expressed with regards to the direct translation of some terms and their cultural appropriateness in Greek. The debates were focused on the terms: shortness of breath, lack of energy, poor (appetite/mobility), feeling depressed and information. (See Table 4)

**Table 4:** Conceptual definition consensus

Shortness of breath	The exact translation would be *‘κοντανασαίνω’, ‘λαχάνιασµα’, ‘δύσπνοια’*. However previous experience had shown that patients understand better the term ‘*δυσκολία στην αναπνοή’* which would be translated as ‘difficulty in breathing’ in English.
Lack of energy	Originally translated as ‘***έλλειψη ενέργειας*’**. However, there were suggestions to use the term ‘*ενεργητικότητα*’ as a possible alternative, defined as the action performed if the person has energy.
Poor (appetite/mobility)	The term ‘poor’ cannot be translated word for word into Greek. The term ‘***µειωµένη όρεξη’*** or ‘***περιορισµένη κινητικότητα’*** which means ‘reduced’ and ‘limited’ are more lay Greek expressions for poor appetite and poor mobility respectively.
Feeling depressed	This term for depression initiated a lot of discussion since the term depression points to a medical diagnosis, similar to anorexia. The medical term encompasses not just the element of sadness, which patients have, but also the element of apathy which is not present. Hence the term *‘****νιώθω θλίψη’*** which is equivalent to ‘feeling sad’ was thought to be more appropriate. However, no consensus was reached for this term.
Information	The proposed term for information was ‘*πληροφορίες*’. However, clinicians pointed that within the context of health care (related to diagnosis, prognosis) often the term ‘***ενηµέρωση***’ (updating) might be more accurate and more frequently used by clinicians.

#### Phase II: Forward translation

There were grammatical and content differences in the first translation stage, regarding item terms, questions phrasing as well as the Likert response categories. Dissimilar translations were found in 6 items: item 3 ‘lack of energy’, item 6 ‘poor appetite’, item 8 ‘sore or dry mouth’, item 10 ‘poor mobility’, item 11–12 ‘have you been feeling’, item 14 ‘at peace’ and item 17 ‘problems’. The Likert scale responses that had alternative translations were found in question 2: (‘slightly’, ‘severely’, ‘overwhelmingly’) and question 3–8 (‘occasionally’, ‘most of the time’, ‘always’). Question 2 was found to have grammar and syntax differences with regards to tense, length of sentence and request phrasing (imperative).

The different roles of the two translators (doctor/ lay member) proved very useful, as they provided different insights. The clinician highlighted the terms used in everyday clinical practice, whilst the lay member provided better options (lay friendly) with general understanding of instructions and question formulation. In addition, we consulted a linguist with regards to certain grammar and syntax discrepancies, to confirm clarity of the instructions, as well as semantic equivalence with regards to synonym terms.

Considering the literature review and the subsequent interviews, we reached consensus with the forward translation version. The forward translations highlighted the differences identified during phase I and were resolved during discussions with translators and translator mediator. It resulted in semantic modifications for the 3rd, 6th, 10th and 14th items. Specifically, the term ‘unconscious’ (διαταραχή συνείδησης- staff version) was also modified to reflect its specificity in Greek.

Despite the more complex forms, we agreed to include both gender options (e.g., s/he), opting for gender- inclusive language.

#### Phase III: Backward translation

Both backward translations did not reveal significant discrepancies when compared to the original IPOS tool. However, there were some differences in the backward translation, specifically with regards to verb tenses and synonym choices, rather than the direct translation of words. Differences were found in 3 items: ‘feeling depressed’ (back translated as feeling distressed/sadness), ‘feeling anxious’ (back translated as feeling anguish/worried) and poor ‘appetite/mobility’ (back translated as anorexia- loss of appetite). Another synonym was used specifically for mobility. The Likert response ‘overwhelmingly’ was back translated as ‘unbearably’, whereas slightly as mild. The reported dissimilarities in the above terms, already identified as holding some challenges in previous phases, were further discussed with the two translators and the moderator to identify the best equivalent.

#### Phase IV: expert review

The expert review discussions focused on both item terminology, language formality and Likert scale responses, which remained unresolved. Discrepancies in the backward translations for anxiety, depression and poor (appetite/mobility) were also discussed and agreed to further explore in the following phase. Specifically, further discussions were necessary for the term ‘shortness of breath’, which was translated to ‘difficulty in breathing’ (Δυσκολία στην αναπνοή). Similarly, the term ‘Poor mobility’ was debated between synonymous terms (µειωµένη/περιορισµένη κινητικότητα) and was agreed to test both terms at the cognitive debriefing phase. The term ‘poor appetite’ was translated as either ‘anorexia’ (ανορεξία) or ‘reduced appetite’ (µειωµένη όρεξη), with the latter term being agreed as friendlier but also less confusing (clinical diagnosis). No consensus was reached for the term ‘feeling at peace’, with different terms being suggested to be explored during the cognitive debriefing phase (ειρηνικά, ειρήνη µέσα σας, ψυχική ηρεµία). The term ‘unconscious’ (staff version- grading) created ambiguity. The translators and mediator considered the exact translation of the term in Greek,“αναίσθητος”, but the expert committee argued that the term is used for anaesthetized patients more often and thus was not appropriate. They instead suggested the term “consciousness’ disruptions” (διαταραχές συνείδησης) as per phase II, although consensus was not agreed and thus both terms were referred to the next phase testing.

A debate with regards to use of appropriate tense highlighted the need to clarify if items refer to an incident in time, or a continuous state. Similarly, discussions regarding Likert responses focused on identifying the subtle changes in between the options, so that grading between options becomes more obvious.

Question and instruction formulation was also debated within the expert review team, considering either the singular(informal) or plural (formal/polite) form, often used in Greek. The team agreed to a simple, but rather formal language, so that the tool addresses patients in a polite but clear manner. Lastly, question 9 on practical problems was reviewed to a better grammatical form in Greek (comment by the linguist).

#### Phase V Cognitive debriefing

The cognitive interviews resulted in revisions of 5 out the 17 items of the translated IPOS tool (Table 5). Both focus group interviews showed that most questions and answer options worked well for the majority of both patient and professional participants. Reported difficulties were focused mainly on comprehension and a few concerned judgements. No problems were reported with retrieval and response formulation. The comprehension and judgement challenges identified in the pre-final version were successfully resolved in the cognitive interviewing phase. The results are presented under comprehension, judgement, and acceptability. The interview results and the changes made based on them are shown in detail in Table 6.

**Table 5:** IPOS changes after cognitive Interviews Final CHANGES

Items	Terms in the English version	Revised
	What have been your main problems or concerns over the past 7 days?	No
	Below is a list of symptoms, which you may or may not have experienced. For each symptom, please tick one box that best describes how it has affected you over the past 7 days.	Yes
1	Pain	No
2	Shortness of breath	No
3	Weakness or lack of energy	Yes
4	Nausea (feeling like you are going to be sick)	No
5	Vomiting (being sick)	No
6	Poor appetite	No
7	Constipation	No
8	Sore or dry mouth	No
9	Drowsiness	No
10	Poor mobility	No
	Please list any other symptoms not mentioned above, and tick one box to show how they have affected you over the past 7 days.	Yes
	Over the past 7 days:	
11	Have you been feeling anxious or worried about your illness or treatment?	No
12	Have any of your family or friends been anxious or worried about you?	No
13	Have you been feeling depressed?	Yes
14	Have you felt at peace?	Yes
15	Have you been able to share how you are feeling with your family or friends as much as you wanted?	No
16	Have you had as much information as you wanted?	Yes
17	Have any practical problems resulting from your illness been addressed? (Such as financial or personal)	Yes
	How did you complete this questionnaire?	No

**Table 6:** Issues regarding IPOS completion identified in the cognitive debriefing phase

IPOS item (prototype)	IPOS answer options (prototype)	Aspects identified with supporting quotations	Revised IPOS item after focus group	Revised IPOS answer options after focus group
Q1. What have been your main problems or concerns over the past 7 days? (P) What have been the patients’ main problems over the past 7 days? (S)	Open questions with three lines for answers	Good comprehension (14/15) of the question. Participants welcomed the term ***προβλήµατα*** and perceived it as a broader term than symptoms, which can include concerns and family issues. *…I think that ‘problems’ is the right word, because it does not refer only to symptoms, it can be other things, such as things that have to do with the family or the carer (S-3)* *I was thinking of the patient as a patient, but also the family’s problems. All the problems in this household (S-2)* *Yes, it is very simple/clear. For me is the fear of the disease (P-1)* *You mean the 3 major issues because I have many, not just 3. It is the colostomy management, …, family pressure, financial issues (P-5)* A few participants suggested to include the phrase ‘**that bothered you in particular’** to help respondents to prioritise their key problems. *Can you add the phrase: that they particularly bothered you, to make it clear that you want the ones at the top of list? (P-3)*	No changes to staff version What are the main problems or concerns **that particularly bothered you** over the past 7 days (P)	Remains an open question with three lines for answers
Q2. Bellow is a list of symptoms, which you may or may not have experienced. For each symptom, please tick one box that best describes how it has affected you over the past week (P) Please tick one box that best describes how the patient has been affected by each of the following symptoms over the past 7 days (S)	Not at all, Slightly, Moderately, Severely, Overwhelmingly Cannot assess (e.g., unconscious) (S)	Good comprehension (10/15). 4 participants found the question too long and wordy. *In order to answer this question, I had to read it three or four times, I somehow lost the meaning. I believe this is not the right way to express it, anyway, it is not easy to understand (S-7)* *The question is too long… in the beginning…and we have said it is difficult to grasp its meaning (S-3)* Both patient (5/6) and HPs (5/9) participants revealed a confusion of the meaning of the question, as to whether it assessed severity or impact (how versus how much). *It asks about the grade of the following symptoms (P-3)* *Here it talks about the intensity… How much it has affected the patient. I think this has to do with both the intensity but also how it might have changed their ability to function… not just the grade… It includes many parameters, not just the severity of the symptom (S-3,4)* *To be honest, I have been carried away by some of the symptoms and considered more the intensity rather than the impact (P-7)* *When talking about impact, I think it is a composite of many factors, such as severity, intensity, length of persistence, repetition pattern, influence on functionality. (S-3)* *It is matter of grade (P-4)* All HP participants found answer options clear and grading sufficient, with the exemption of the example of the last one (i.e., unconscious/ αναίσθητος). The term unconscious was reported to have alternative meanings: being insensitive, or indifferent (3/9) or somebody under anaesthesia (1/9). *Unconscious does not really apply to me; it refers to operating theater (S-8)* *I think that the word “unconscious”, in Greek culture may also have a different significance, meaning you are indifferent… (S-9)* A phrase that describes level of consciousness (4/9) or ability to communicate (3/9) was proposed. Overall consensus was on level of consciousness. *Compromised level of communication, for whatever reason, …, low level of communication(S-3)* *confusion or confused, something like that that we use in our everyday practice (S-8)* *Perhaps we could use loss of consciousness (S-8, S-7)* Patients found challenging the rating of symptoms when those fluctuated during the measured period. (See more on symptoms bellow)	Here is a list of symptoms that you may or may not have experienced. For each symptom, please choose the corresponding box (**only one**) that describes best how it affected you over the past 7 days (P) Please choose the box (that describes better how each of the following symptoms affected the patient over the past 7 days (S)	Not at all (Καθόλου) Slightly (ήπια) Moderately (µέτρια) Severely **(σοβαρά-S, ** **πολύ-P)** Overwhelmingly (ανυπόφορα) It cannot be assessed (e.g., **reduced level of consciousness**) Δεν µπορεί να εκτιµηθεί **(π.χ. απώλεια του επιπέδου συνείδησης**)
Pain		Good comprehension by all participants Some patients (3/6) found it hard to judge the severity of pain and how it affected them, as it fluctuated over the 3 or 7 days. They solved this problem by estimating a mean value over the three days.	Pain (Πόνος)	
Shortness of breath		Good comprehension by all participants. A debate between the terms dyspnea and difficulty-in-breathing led to a consensus towards the latter term. The term difficulty in breathing (=*Δυσκολία στην αναπνοή)* was understood as a better description of dyspnoea regardless its cause (e.g., physical, psychological) *I prefer the difficulty in breathing as it describes the feeling of dyspnoea, however it might be experienced by the patients (e.g., due to disease progression, depression, panic attack (S-4)* *… If refers to breathing, if it is unobstructive or not. For whatever reason…and whatever feeling it is expressed (S-3)* *Difficulty in breathing might mean air is cut short (S-8)) the air is not enough (P-3) I cannot take a deep breath (P-4)*	Difficulty in breathing (Δυσκολία στην αναπνοή)	
Weakness or lack of energy		Good comprehension of the word weakness (=Αδυναµία) (15/15). Participants explained it as a term describing tiredness, and exhaustion. *When I say weakness, I can think of fatigue… I can think of exhaustion (P-1, P-3)* *Thinking of patients, they usually say, I feel tired, hence it seems weakness has to do with feeling fatigued (S-6). * *Weakness also refers to mental or spiritual fatigue, not being in the mood, feeling low (P-3)* There was a debate about the phrase lack of energy (= Έλλειψη ενέργειας/ ενεργητικότητας). Professionals favored it (8/9) and considered it as a synonym to weakness (S-3); or related to vigour (= preferred term in Greek: ενεργητικότητα) (S1, S3, S4, S7) and some connected it to activities performance (δραστηριότητες) (S-4, S-8)) *Weakness is not enough; lack of energy refers to the activities the patients used to do etc. (S-8)* *I thought of the activities the patient reports over the last few days… if he had to reduce his activities because he felt tired…(S-4)* Patients preferred the Greek term (**ενέργεια**) for energy (6/6) Lack of energy was then described as not feeling like doing anything, or not willing to leave the chair. *Lack of energy (ενέργεια) is broader than energy (ενεργητικότητα), I prefer the first one (P-1). What can I say, there are days I do not want to hear anyone (P-6)* *I do not have energy means I feel like doing nothing, not feeling the need to move from the sofa, or going out (P-3).* *Lack of energy means even if I want to go for a walk, I don’t feel like I can…, feeling weak or feeling low (P-2)*	Weakness or lack of energy (Αδυναµία ή Έλλειψη ενέργειας)	
Nausea (feeling like you are going to be sick)		Good comprehension by all participants and overall consensus (15/15). Participants welcomed the explanation in the brackets as a helpful description. *I like the explanation in the brackets. Maybe nausea is enough for staff, but for some patients the explanation might be useful (S-5).*	Nausea (Ναυτία) Feeling like you are going to be sick (Τάση για έµετο)	
Vomiting		Good comprehension by all participants, overall consensus (15/15). Difficulties with the rating were reported, as some participants tried to rate the severity of the symptom, not its impact.	Vomiting (Έµετος)	
Poor appetite		Good comprehension by all participants. The term poor appetite (=Μειωµένη όρεξη για φαγητό) was preferred to anorexia. As appetite might be connected to many things in Greece, the word ‘for food’ was agreed to be added, for clarity. *I don’t feel like eating anything (P-2)*	Poor appetite (reduced appetite for food: Μειωµένη όρεξη για φαγητό)	
Constipation		Good comprehension by all participants (15/15) *This is a common symptom that really affects patients, and they often mention it even when you don’t ask (S-8)*	Constipation (Δυσκοιλιότητα)	
Sore or dry mouth		Good comprehension and agreement to both terms by HPs (8/9). Terms considered to be inclusive of overall mouth problems from dry mouth to inflammation and infection. Yet, patients gave examples referring to dry mouth only (3/6) *I like the term ‘sore mouth’ as it can include many problems, such as ulcers inflammation, and stomatitis (S-8)* *Sore mouth might include symptoms related to mucositis, ulcers, etc. (S-4)* *For dry mouth… I need to always have a glass of water next to my bed, otherwise the suffering is unbearable(P-4)* *My mouth and my tong are dead, so dry that I cannot even swallow my saliva (P-2)* *My tong and my lips are so dry that I feel them numbed(P-5)*	Sore or dry mouth (Ερεθισµένο ή ξηρό στόµα)	
Drowsiness		The term was understood well by most participants (13/15). It was related to feeling sleepy and was measured against its impact on everyday life. *Patients describe drowsiness as feeling sleepy which affects their everyday life (S-2)* *When they feel sleepy and cannot respond the activities of their daily life (S-4)* *I have the opposite, insomnia stress and cannot sleep (P-2)*	Drowsiness (Υπνηλία)	
Poor mobility		Good comprehension and consensus on the term by all participants (15/15) *I like this as it makes clear that mobility is different from energy (P-5) For me it is difficulty with walking, ability to reach out for things (P-1).*	Poor mobility (Περιορισµένη κινητικότητα)	
Please list any other symptoms not mentioned above and tick one box to show how they have affected you over the past 7days. (P) Please list any other symptoms and tick one box to show how you feel each of these symptoms has affected the patient over the past 7 days (S)		Good comprehension and consensus achieved by all participants after some minor changes in the phrasing of a few words in the question. Both versions were suggested to use past tense. Patient version kept the ‘tick a box- only one’ whilst staff version agreed to ‘choose a box’. *To make the question concise, professionals suggested to replace the ‘any other’ with ‘additional’. (S-2)* Patients found the question useful as it gave the opportunity to add further symptoms but also break down symptoms reported above. *I wrote symptoms not mentioned above (P-3)* *I wanted to be more specific with regards to poor mobility. It has to do with my left arm, so I added here(P-1)*	Please list any other symptoms **you had** and not mentioned above and tick **the box (only one)** that describes better how they have affected you over the past 7 days. (P) Please list any **additional** symptoms and **choose** a box to show how you think each of them has affected the patient over the past 7 days (S).	
Q3. Over the past 7 days, have you been feeling anxious or worried about your illness or treatment? (P) Over the past 7 days, has s/he been feeling anxious or worried about his/her illness or treatment? (S)	Not at all, occasionally, sometimes, most of the time, always Cannot assess (e.g., unconscious)	Good comprehension, overall consensus by all participants. Two HP respondents (S-4,6) wished anxiety was not restricted to disease or treatment only. *It somehow narrows the scope and often it is more than treatment and disease that patients feel anxiety (S-6) What if the patient is anxious about other issues, beyond the disease and treatment. Do we still score for it? (S-4)* The grade of response was discussed. The difference between occasionally and sometimes was found unclear to 3 HPs and 2 patients (5/15). ‘*Occasionally*’ was originally translated as ‘*Περιστασιακά’* and changed to ‘*λίγες φορές’*, for staff version, in concordance with the patient version. ‘Always’ was originally translated as ‘*πάντα’* and changed to ‘*συνεχώς*’ for both version. Cannot assess (e.g., unconscious) changed to e.g., ‘reduced level of consciousness’, following responses to Q2.	Over the past 7 days, have you been feeling (=**νοιώθατε**) anxious or worried about your illness or treatment? (P) Over the past 7 days, has s/he been feeling (=**ένοιωθε**) anxious or worried about his/her illness or treatment? (S)	Not at all (Καθόλου) Occasionally (**λίγες φορές)** Sometimes (αρκετές φορές) most of the time (τις περισσότερες φορές) (P, S) always (P: πάντα, S: **συνεχώς)** It cannot be assessed (e.g., **reduced level of consciousness- ‘απώλεια επιπέδου συνείδησης’)** (S)
Q4. Over the past 7 days, have any of your family or friends been anxious or worried about you? (P) Over the past 7 days, have any or his/her family or friends been anxious or worried about the patient? (S)		Good comprehension by all participants. Variation in assessing anxiety of different members of family/ friends. Professionals argued of not having access to patient’ friends unless directly involved in their care (4/9). *Not sure the relevance of friends, unless involved in their care (S-9)* *Cannot assess the friends’ network of the patient(S-2)* Also, hard to quantify for a group of people, especially if there is variation at anxiety levels (3/9). Patients could identify those important to them- not dilemma on who to consider (5/6). *… are we talking about the children, or the main carer? Who to consider?… children might be more sensitive and thus more anxious, comparing to a spouse. (S-6) Do you mean the nuclear family or the broader family here? (S-8)*	Over the past 7 days, have any of your family or friends been anxious or worried about you? (P) Over the past 7 days, have any or his/her family or friends been anxious or worried about the patient? (S)	
Q5. Over the past 7 days, have you been feeling depressed? (P) Over the past 7 days, do you think s/he felt depressed? (S)		Patients reinforced the chosen Greek terms for ‘feeling depressed’ (= Νιώθατε θλίψη) instead of the word ‘κατάθλιψη’. Different synonyms were discussed (λύπη, στεναχώρια) but the original term was perceived as conceptually broader, encompassing different dimensions (5/6). All professionals agreed to the term (9/9) *‘Sadness, mixed with stress shape the ‘feel depressed’; (P-3)* *Feeling depressed might also include a sense of isolation and subsidence (P-2)* *It is the feeling that compromises joy…and your tranquility (P-5)* *…It is when you are sad, feeling in a tide place, feeling suppressed… the feeling of not having enough energy to cope (P-6)*		No changes made. Greek version: ‘Νιώθατε θλίψη;’ (P) ‘Νοµίζετε ότι ένιωθε ο/ή ασθενής θλίψη’; (S)
Q6. Over the past 7 days, have you felt at peace? (P) Over the past 7 days, do you think s/he felt at peace? (S)	Always, Most of the time Sometimes Occasionally Not at all (P, S) Cannot assess (e.g., unconscious) (S)	The social spiritual or emotional context of the Greek word peace (=ειρήνη) was discussed, and agreed to the meaning of tranquility/ serenity. (13/15) Two HP preferred the word ‘ειρηνικά’, but some patients highlighted its relevance to religion terminology and thus not relevant to all. *‘… when the priest wishes for peaceful ending of life… internal piece’ (P-5)* *‘Feeling at peace is often used in church, not appropriate for everyone (S-3)* *‘I prefer the inner serenity (ψυχική ηρεµία), it reflects better the inner state of peace, because being at peace might relate to the social context, peace with others.’ (P-3)* *I like more the feeling calmness, internal serenity, quietness, tranquility, not so much feeling peacefully (P-6)*	Over the past 7 days, have you felt **inner serenity (**Νιώθατε **ψυχική ηρεµία)? (P)** Over the past 7 days, do you think s/h felt **inner serenity (**Νοµίζετε ότι ένοιωθε **ψυχική ηρεµία)? (S)**	Always (**συνεχώς**) Most of the time (τις περισσότερες φορές) Sometimes (αρκετές φορές) Occasionally (**λίγες φορές**) Not at all (Καθόλου) (P, S) It cannot be assessed (e.g., **reduced level of consciousness- απώλεια του επιπέδου συνείδησης’)** (S)
Q7. Over the past 7 days, have you been able to share how you are feeling with your family or friends? (S) Over the past 7 days, has the patient being able to share how s/he is feeling with his/her family or friends as much as s/he wanted? (S)		Good comprehension by all participants and examples shared to illustrate the importance of the question. Participants expressed a difference of importance between family and friends. Some patients objected the reference of family and friends as of equal alternatives. (3/6) *‘First comes the family and then friends. Why using or?’ (P-4)* *‘Family and friends are not the same thing. I wouldn’t put them in the same question’ (P-2)* *‘… how about phrasing it: “share your feelings with your family and maybe your friends”, to give priority to the family’ (P-3)*	Over the past 7 days, have you been able to share how you are feeling with your family or friends? (S) Over the past 7 days, has the patient being able to share how s/he is feeling with his/her family or friends as much as s/he wanted? (S)	
Q8. Over the past 7 days, have you had as much information as you wanted? (P) Over the past 7 days, has the patient had as much information as s/he wanted? (S)		The term ‘information’ **(= πληροφόρηση)** was debated in comparison to term ‘briefing/ updating’ **(= ενηµέρωση).** Consensus reached for the term ‘ενηµέρωση’, given that it is the common term used in clinical practice. (9/15) *‘I think updating(* ***ενηµέρωση*** *) is better than information’ (* ***πληροφόρηση*** *) (P-2) … I agree with this, maybe it is. (S-8)* *‘We usually invite patients and families for ‘* ***ενηµέρωση*** *’, not ‘* ***πληροφόρηση*** *’. We don’t use this term in the clinic.’ (S-3)* Clarity was requested with regards to being informed ‘by whom (6/15)’ and ‘about what’ (5/15). Patients included families as being information givers (4/6). *‘ As for information, I thought of medical issues, but also more general info such as the team, the service, who we are, psychosocial resources, etc…’(S-9)* *‘When we say information, do we mean by the doctors or also our family? (P-1) Correct, information comes from your social environment as well (P-4).* Patient participants (3/6) found it difficult to rate satisfaction with information, as information level may vary according to type of information. *‘Let’s say, I put occasionally because I was happy with treatment information, but not so with illness progress, or other matters.’ (S-9) ‘I agree, I did not have enough info re medical issues by my doctor, but I was happy with the social worker about psychosocial matters’ (S-4)*	Over the past 7 days, have you had as much **information** as you wanted (Τις τελευταίες 7 ηµέρες είχατε τόση **ενηµέρωση** όση θα θέλατε? (P) Over the past 7 days, has the patient had as much i**nformation** as s/he wanted? (Είχε ο ασθενής τόση **ενηµέρωση** όση θα ήθελε;) (S)	
Q9. Over the past 7 days, have any practical problems resulting from your illness been addressed? (Such as financial or personal) (P) Over the past 7 days, have any practical problems resulting from his/her illness been addressed? (Such as financial or personal) (S)	Problems addressed/ No problems (P, S) Problems mostly addressed (P, S) Problems partly addressed (P, S) Problems hardly addressed (P, S) Problems not addressed (P, S) Cannot assess (e.g., unconscious)	The proposed term for problems was debated and agreed to change from ‘*ζητήµατα (= matters)* to ‘προβλήµατα’. Most participants thought it was clearer and corresponded better with the answer options (10/15). The offered example in brackets was valued for clarity by all. The responses formulation and grading seem to have some issues: The first response was found by some people confusing, given that it incorporated two different responses. The recommended that πλήρως (= fully) καθόλου (= not at all) to be included in the grading of the answer options. Debate about the grading of the response. There was a consensus that the first option included two answers as one option. Participants thought this might be confusing and suggested alternative expressions that were more descriptive (8/15). Some words changed in the grading, to make the grade of the options more distinctive. Some variation between staff and patient options was recorded. *‘The problems were fully addressed’ / ‘there were problems and were resolved’ (S-1,8,4) ‘Problems were addressed’ (P-5,3,6)* *‘… no problems’ (S-1,8,4); ‘there were no problems’ (S-1,2,4,7; P-2,5,6)*	Over the past 7 days, have any practical **problems (**=προβλήµατα**)** resulting from your illness (νόσο σας) been addressed (such as financial or personal)? (P) Over the past 7 days, have any practical **problems** (**=προβλήµατα**) resulting from his/her illness (=την νόσο του/της) been addressed? (Such as financial or personal) (S)	Problems were addressed/ **There were no problems** (Τα προβλήµατα αντιµετωπίστηκαν/ **δεν υπήρχαν** προβλήµατα) (P) Problems were **fully** addressed **/ there were no problems. (**Τα προβλήµατα **αντιµετωπίστηκαν πλήρως / δεν υπήρχαν** προβλήµατα) (S) Problems were **largely** (**=σε µεγάλο βαθµό)** addressed **(**P, S) Problems **partly (= µερικώς)** addressed (P, S) Problems **hardly** (=**µόλις που)** addressed (P, S) Problems not addressed **(at all = καθόλου) (S)** Cannot assess **(e.g., reduced level of consciousness)** (S)
Q10: How did you complete this questionnaire (P)	On my own With help from a friend or relative With help of from a member of staff	No problems or concerns with Q10	How did you complete this questionnaire (P)	On my own With help from a friend or relative With help of from a member of staff

P: Patient, S: Staff, HP: health professional

Changes made to IPOS questions or answer options after cognitive interviews are typed **in bold**

### Comprehension

Comprehension difficulties were identified in both patient and health care professional interviews. Participants welcomed the term problems (προβλήµατα) and perceived it as a broader term conceptually, compared to symptoms.  Nevertheless, they reported difficulty in distinguishing them and listed them interchangeably in Q1 and Q2b a few times. We, thus, decided to rephrase the first question, highlighting the troubling nature of the problem/ or concern for the patient. We also changed the phrase ‘*any other symptoms*’ to ‘*any additional symptoms’* in the staff version, to highlight that the question is looking for any further symptoms in relation to the 10 items in previous list (Q2a), per their request.

Pain, shortness of breath, nausea, vomiting, poor appetite, mobility drowsiness, sore or dry mouth and constipation listed in question 2 were well understood by all participants. Comprehension difficulties were identified with the terms: lack of energy (Q2)/ feeling depressed (Q5)/ feeling at peace (Q6).

#### Lack of energy

Two synonyms (Αδυναµία ή έλλειψη ενέργειας) received interchangeable meaning by different participants. Weakness or lack of energy referred to physical activity/ mobility by some participants, whilst for others it referred to a state of mental or psychological languor. Inclusion of both terms satisfied all participants.

#### Feeling depressed

Two Greek terms were discussed with patients that translate to feeling depressed. Patients preferred the choice of the word ‘θλίψη’ instead of ‘λύπη’ (feeling sad). Participants expressed the view that ‘feeling sad’ is different from ‘feeling depressed’ and provided different dimensions of it (5/7). Hence the term ‘θλίψη’ was adopted.

#### At peace

Some patients (3/6) connected the Greek term ‘ειρηνικά’ to inner peace as identified by the orthodox Christian religion; and some with specifically a pray for peaceful ending. This led some participants associating this term with dying and death. The Greek term for serenity (ψυχική ηρεµία) was accepted by all participants (6/6) which was discussed in the context of acceptance of the situation.

*It* *is the feeling that compromises joy, tranquillity, internal quietness (P-5)*

*I like more the feeling calmness, internal serenity, quietness, tranquility, not so much feeling peacefully. (P-6)*

#### Information

Participants debated about the term ‘πληροφόρηση’ (information) versus the term ‘ενηµέρωση’ (briefing/ updating). They seem to prefer the term ενηµέρωση, as oftenused in clinical practice.

*Updating, it would be better to say updating… I agree with this, maybe it is. (S-8)*

Patient participants requested clarity with regards to ‘*who’* provides the information (6/7) and ‘*about what’* (5/6). Patients included families as being information givers and considered information coming from different resources. They were suggested to consider all types of information coming from the health care team.

*‘As for information, you mean medical update, general info, psychosocial support, benefits, …?’(P-3)*.

*‘When we say information, do we mean by the doctors or also our family? (P-1)*

*‘… information comes from your social environment as well, what information you mean?’ (P-4).*

#### Practical problems

There were some issues with this item. The term ‘ζητήµατα’ (matters) that was chosen in previous phases, was suggested to be replaced by the term ‘προβλήµατα’ (problems). Most participants (10/15) considered it more specific and better corresponding with the response options.

Also, the response options for this item(Q9) were found to be confusing by some participants (8/15), as it incorporated two different responses. They, thus, recommended the terms πλήρως (fully), καθόλου (not at all) to be included in the grading of the answer options. With regards to the option ‘being unable to assess’and the example given (e.g., unconscious), staff participants suggested the term ‘reduced level of consciousness’, with two conceptually equivalent terms (έκπτωση/ απώλεια επιπέδου συνείδησης). It was agreed to keep the second option, as commonly used within clinical contexts.

### Judgement

Some issues were reported with the structure of question 2. Some participants rated their symptom severity and not the impact. Severity of symptoms versus impact was a common difficulty (instructions of Q2) for both patient and health-care participants. Restructuring the sentence, emphasizing the focus on impact, has helped with clarifying this confusion.

*The truth is that I scored thinking of severity and not of how much it affected the patient. Can you make this clearer? (S-7)*

The time window **“***Over the past 7 days’* was considered too short for patients in the day care setting, but too long for some patients in the home care service. Choosing the appropriate version (3- or 7-days’ time interval) for patient assessment, was added in the tool instructions.

Another problem was the presence of fluctuating symptoms. Fluctuating symptoms resulted in difficulties with judging severity and impact. Patients had to rate symptoms intensity that could have had fluctuations and different impact on their experience over the past 7 days. This caused difficulties in choosing an answer. A solution was agreed to o report the mean value and not the highest intensity in the case of fluctuating symptoms. A clarification was added in the instruction manual.

*I only feel breathless when I am tired. But it does not give me this option; it says not at all, slightly, etc. when I seat, I am not breathless. if I walk more then I feel it… what should I choose? (P-6)*

With regards to response options in Q2, we chose different option responses for patient (severely = πολύ) and staff versions (severely = σοβαρά), following their preferences.

With regards to questions 7, participants found hard to answer about sharing feelings with friends, arguing that professionals don’t have access to patients’ friends, unless they are directly involved in the patient care. Equally, they struggled with quantifying for a group of people (if more than one family member was involved) in question 4, especially whenvariation on anxiety levels was assessed among them.

*… I think spontaneously that this question refers to some member of the family, naturally children can be part of the family and they can be more sensitive than the carer (S-9)*.

*Do you mean exclusively the core family members and not a broader circle of people? (S-8)*

With regards to response in Q8, some patients (3/6) found difficult to rate satisfaction with information, highlighting variability between different areas of provided information.

*‘Let’s say, I put occasionally because I was happy with treatment information, but not so with illness progress, or other matters.’ (S-9) ‘I agree, I did not have enough info re medical issues by my doctor, but I was happy with the social worker about psychosocial matters’ (S-4)*.

### Acceptability

The Integrated Palliative Care Outcome Scale was well accepted by both patients and health professionals. Patients and health-care professionals felt that the content and format of IPOS was appropriate, feasible, and not burdensome. Time of patients to complete the IPOS tool ranged from min 7.7 to max 11.24 min. All questions were considered important and none as inappropriate.

Most health care professionals stated that the questions were similar to those already usingin their clinical practice. Nevertheless, the tool provided them with a more structured approach into patient assessment. Patients suggested that some of the questions were very helpful, as they served as a guide to self-assessment (especially the symptom list question- Q2). The questions referring to psychosocial needs (Q3, Q4, Q5 and Q6) were welcomed as very important by patients, as they offered an opportunity to share their more difficult and personal concerns and feelings.

### Layout concerns

Difficulties in reading and filling the forms were reported by participants. Some layout changes were made to make the tool easier to complete, such as increasing the types of fonts and widening the interline space.

Question 2 was reported to be difficult to process, due to its length. The sentences were rephrased and shortened with good acceptability.

*…. In order to answer this question [Q2], I needed to read it three to four times. I was lost. (S-7)*

*The question is too long…it is difficult to grasp the meaning (S-3)*

## Discussion

This study translated and culturally adapted the IPOS (patient & staff) tool for Greek patients. The 6-phase process of cross-cultural adaptation allowed for the development of a Greek version of IPOS and demonstrated face and content validity and acceptability. Both patients and health care professionals (HCP) involved in the focus group discussions thought the questionnaire was appropriate, feasible and easy to use in everyday clinical practice and none withdrew from participating in the study. Consistent with previous published work, professionals highlighted the tool’s advantage in clinical assessments and exploration of sensitive areas, such as depression, spiritual distress and family support [25–27].

The challenges of cross-cultural adaptation between the two languages and cultures were revealed early on at the translation phases, both in the wording of questions but also when looking for the conceptual equivalent of certain terms.. The most important linguistic cultural differences between Greek and English were found in the formation of instructions/questions. We must agree with the French group that translating metaphors and expression to other languages is difficult and the concept behind the words has to be discussed and agreed upon [27]. In our study, the linguist’s support in refining the wording, length and structure of the first two questions (Q1-Q2) was significant. We would encourage the consideration of such role in the cultural adaptation of tools in different contexts and languages. 

The terms ‘lack of energy’, ‘poor appetite’, ‘shortness of breath’, ‘poor mobility’, ‘feeling depressed’ and ‘at peace’ were offered different translation versions. Lack of energy was translated to ‘lack of vigor’ (ενεργητικότητα) or spirit (διάθεση) or weakness (αδυναµία), highlighting the different dimensions of the concept. Particularly, ‘weakness’ and ‘lack of energy’ was discussed for its interchangeable meaning in Greek. Both terms were tested at the cognitive debriefing phase and agreed to be included in the final version, as in other translations [25, 28].

‘Shortness of breath’ was translated to either ‘dyspnea’ (δύσπνοια) or ‘panting’ (λαχάνιασµα/ κοντοανασαίνω). The different backgrounds of translators (health professional/ lay member/academic) chose terms related to their experience, understanding of the concept, and exposure to existing clinical terminology. Terminology experts supported the conceptual dialogue of terms, as used in other assessment tools.

Τhe word ‘poor’, used to characterize appetite and mobility in the tool, was also difficult to translate, on a semantic level, as in the French version [25]. In order to describe the limitations in appetite and mobility we agreed on the term ‘limited’ (περιορισµένη) for mobility and ‘limited’ (µειωµένη) for appetite. As for the term ‘appetite’, we opted for the more descriptive form ‘desire for food’, as the Greek term for appetite has a broader meaning (desire= όρεξη).

Despite the detailed approach in forward and backwards translations, the patients’ and clinicians’ perspective proved to be vital when refining the translated tool at the cognitive debriefing phase. Particularly patients in the focus groups suggested terms that have been excluded at the translation phase and introduced semantic insights that the research team had not considered before. The different perspectives proved valuable as they challenged common biases associated with used terms, which are instrumental for reaching content validity.

The term ‘at peace’ (ειρηνικά) generated most of the controversy in the focus groups discussions. Similarly, to other IPOS translation groups, the term was debated for its meaning, with different explanations alluring to social, psychological or spiritual dimensions [26, 29]. However, the cultural links of this word with the Greek orthodox church and death and dying blessings, generated discomfort to some participants, highlighting the importance of cognitive debriefing within cultural adaptation process in specific contexts [22, 30]. In order to maintain the spiritual dimension of the term, the groups favored the term ‘serenity’ (ψυχική ηρεµία = state of soul calmness).

In contrast with other groups’ experience [25, 28, 29], the Greek term used for ‘feeling depressed’ (θλίψη), was well accepted. As the clinical term depression (κατάθλιψη) had been already eliminated at prior stages, the adopted term- with a closer meaning to sadness- was perceived appropriate. Different synonyms were discussed (στεναχώρια, λύπη, θλίψη). The team adopted the term that generated richer accounts, describing a feeling of sadness; a feeling of compromising joy and internal quietness; a sense of being squashed.

The expression “problems being addressed”, which was a matter of controversy in the literature [29], was not met with comprehension challenges in the Greek version. The Greek term (τα προβλήµατα αντιµετωπίστηκαν) illustrates both evaluating and solving a problem and was well accepted by all.

Similarly, we did not face any issues with question 3 and its potential binary meaning (‘feeling anxious or worried about illness or treatment), compared to reports by other groups 25, 28. Participants seemed to group the options together and responded to all of them with no conflict.

The debate with regards to grouping family and friends at the question 4(Q4), highlighted a possible cultural reference to family care specific to Greece. Both patients and professional focused group found somewhat the combination of family and friends confusing. They made a distinction between immediate and extended family and friends circles, whilst elaborating on deferent layers of involvement and roles. Consequently, they were troubled with which ones to consider when responding to the question. However, some patients understood the possibility of friendsreplacing their families.; hence we decided to keep both terms, as with the English version.

A similar confusion observed with regards to information givers (Q8), underlined acommunication pattern within the Greek health care culture. Patients stated that medical updates might not necessarily be provided by health care professionals only, but also their families. Hence, they needed further clarification with regards to which provider to consider when responding to the question. This conversation confirms a persistent attitude favoring family-centered over patient-centered decision-making and information disclosure in Greece [31, 32].

Some difficulties were found with judgment during the cognitive debriefing phase. Response options were at times difficult to distinguish and to depict the slight differences in their meaning. As in other groups, “overwhelmingly” was an issue, but also “slight” and “mild” were items of discussion [26, 29]. We tested different synonyms and asked participants to view them in a grading scale, in order to decide on the preferred word, which was proven very helpful. The available options do not reflect semantic differences necessarily, but rather the terminology already used in clinical practice by health care professionals. The term ‘overwhelmingly’ was discussed as ‘unbearable’ (ανυπόφορα) and ‘too much’ (πάρα πολύ). We opted for the ‘unbearable’ option, to ensure of its negative meaning, as per the Swedish group [29].

 The rating of fluctuating symptoms over a period of time, seemed to be a challenge for our participants , something already reported by the Swedish, Italian, German and Estonian study groups [4, 26, 28, 29]. Following existing practice, we agreed to add a note in the instructions to rate the average of severity. The options of 3 or 7 days were also discussed, with participants reporting the time frame as either to short or too long, depending on their illness phase (stable/unstable). We decided to include an instruction note for appropriately choosing the right version- after assessing patient’s condition, similarly to the Swedish study group [29].

### Limitations

The study has several limitations. A limitation is the relatively small sample size (15 participants for the cognitive interview phase). However, small numbers are acceptable for cognitive interviews, as this method is work-intensive and produces rich data [24, 30]. Secondly, all patient participants had a primary cancer diagnosis, which questions the transferability of the findings to people with other limited diseases. However, this reflects the Greek reality, as palliative care is mostly provided within the context of cancer care. Despite the sample limitations, we aimed at recruiting participants from two different palliative care settings, to improve diversity in care experience and disease severity. Although the results reflect the views of the specific patient groups, their complex palliative care needs (being treated in specialist palliative care unit) seem to be an adequate base for the development of a tool that aims to assess palliative care needs from the patient’ perspective primarily.

## Conclusion

This process of developing and considering the various challenges in cultural and linguistic adaptation of the Integrated Palliative Care Outcome Scale **(**IPOS), provided us with an in-depth understanding of how the IPOS tool can be used and interpreted in practice. We translated and culturally adapted the IPOS scale in Greek, maintaining the available four versions for both patients and staff, each with 3- and 7-days recall time accordingly. These versions are now available on the POS website (www.pos-pal.org). Our data suggest that IPOS-Gr has face and content validity and acceptability in the Greek context. Cognitive Interviewing proved valuable in refining concepts, judgement processes and response formulation. The refined version is currently undergoing psychometric validation.

## Clinical implications

The IPOS outcome measure tool is now being used routinely in two oncology / palliative care settings in Athens and it is currently used to evaluate service outcomes in one palliative care service, together with two other PROMs, i.e. Palliative Phase of Illness and Palliative Performance Scale (PPS.) The incorporation in the electronic patient record of the IPOS, as well as the digital display of changes over time, has facilitated training and application of the tool through collective action and reflexive monitoring.

### Electronic supplementary material

Below is the link to the electronic supplementary material.

Supplementary Material 1

## Data Availability

The datasets generated and/or analysed during the current study are not publicly available due the protection of participants’ identities but are available from the corresponding author on reasonable request and following application to the Ethics Committee of Galilee Palliative Care Service.
