# Correction: Translation and cultural adaptation of the Greek integrated palliative care outcome scale (IPOS): challenges in a six-phase process

**DOI:** 10.1186/s12904-023-01312-3

**Published:** 2023-11-22

**Authors:** Despina Anagnostou, Stylianos Katsaragakis, Irene Panagiotou, Elisabeth Patiraki, Aliki Tserkezoglou

**Affiliations:** 1 Kyoto University, Kyoto, Japan; 2 National and Kapodistrian University of Athens, Athens, Greece; 3 National Primary Care, Athens, Greece; 4 Galilee Palliative Care Unit, Spata, Attica Greece

**Correction: BMC Palliat Care 22, 168 (2023)**

10.1186/s12904-023-01278-2

Following publication of the original article [1], the author reported that the given names and family names were interchanged for all authors.

This has been corrected above and the original article has been corrected.
