# Supplementary material for: Translation and cultural adaptation of the Greek integrated palliative care outcome scale (IPOS): challenges in a six-phase process
Source: BMC Palliat Care. 2023 Nov 2;22:168. doi: 10.1186/s12904-023-01278-2 (PMC10621102; doi:10.1186/s12904-023-01278-2)
Supplement: Supplementary file 1 — Supplementary Material 1 [file 12904_2023_1278_MOESM1_ESM.docx]

# Additional File 1.

## Interview guide- Cognitive interviewing phase (adapted by the IPOS resources*)

**Objective:**

To explore the cognitive processes used by respondents when reading, interpreting and responding to items on the IPOS questionnaire.

**Introduction to the focus group interviews, General information about the IPOS tool and process**

- Study purpose, confidentiality, able to stop at any time, decline questions.
- I’m going to show you a questionnaire and I want you to read and answer the questions one at a time.
- We will stop and talk about each question before moving onto the next.
- Please try to ‘think out loud’ as you read and answer the questions (*DEMONSTRATE: that is, When the question is ‘What did you have for breakfast?’, instead of answering ‘Porridge’ I would say ‘Well I was running late for work this morning so I picked up a croissant and a coffee on the way to work, which I ate at my desk’*).
- I will also ask you some more specific things about each question.
- Apologies if the questions get repetitive.
- In this study, we are less interested in your answers to the questions, but *how you arrive* at the answers – what you think the question means, and the things you were thinking about when you chose your answer.
- You can tell me *any* thoughts or views you might have about the questions.

**Focused questions**

*A. General*

1. What were you thinking about when you answered that question?
2. I noticed you hesitated before giving your answer – what were you thinking about then?
3. How long did it take you to complete the questionnaire?
4. How relevant are these questions to patients’ needs according to your opinion?

*B. Comprehension (what does the respondent believe the question to be asking?)*

1. What does the question mean to you, in your own words?
2. What does the word XXXXXX mean to you? (if certain words are thought to be problematic)
3. How easy or difficult was it to understand this question?
4. (If problem) How would you change this question?

*C. Retrieval (could they recall the information required by the question? Was the time frame suitable?)*

1. How well could you remember your experience when answering this question?
2. Was it easy or difficult to think about the past (week) when answering this question?
3. Would there be a different time period that would be easier to understand?

*D. Judgement (is the respondent able to make an evaluation based on the information recalled?)*

1. What were you thinking about when you answered this question?
2. How did you arrive at your answer to that question?
3. Was that easy or hard to arrive at your answer? Why do you say that?
4. How sure are you of the answer to this question?

*E. Response (is the respondent able to map their internally generated answer to a response option?)*

1. How did you choose your answer to this question?
2. Was it hard or easy to select an answer from the options given?
3. Did all options make sense for this question?

*F. Other*

1. Is there anything else you would like to say about this question?/The questionnaire as a whole?
2. Did you find any of the questions upsetting?/embarrassing?/inappropriate?
3. Are there any topics/questions that you would leave out of this questionnaire?
4. Are there any topics/questions that you would add to this questionnaire?
5. Do you have any thoughts about the way your answers were captured? (i.e. tablet/paper)

The Greek topic guide included some additional questions regarding the translation options of the words: anxiety, depression, shortness of breath, lack of energy and felt-at-peace, which were debated in the previous phases.

*Schildmann EK, Groeneveld EI, et al. Discovering the hidden benefits of cognitive interviewing in two languages: The first phase of a validation study of the Integrated Palliative care Outcome Scale. Palliative Medicine. 2016;30(6):599-610. doi:10.1177/0269216315608348
